# Biomechanical stability and monitoring technologies of implant-supported prosthetic components

**DOI:** 10.4317/jced.64338

**Published:** 2026-08-28

**Authors:** Jefferson David Melo de Matos, Mário Alexandre Coelho Sinhoreti, Mauricio Schneider, Robert Dobrin, Daher Antonio Queiroz

**Affiliations:** 1 Department of Biomaterials, Dental Materials and Prosthodontics, São Paulo State University (Unesp), Institute of Science and Technology, São José dos Campos, Sao Paulo, B; 2 Department of Restorative Dentistry, Dental Materials Division, Piracicaba Dental School (FOP UNICAMP), Piracicaba, Sao Paulo, Brazil; 3 Department of Prosthodontics, Nova Southeastern University College of Dental Medicine, Fort Lauderdale, Florida, USA; 4 Department of Restorative Sciences and Public Health Dentistry, Nova Southeastern University College of Dental Medicine, Fort Lauderdale, Florida, USA; 5 Department of Restorative Dentistry & Prosthodontics, UTHealth School of Dentistry, Houston, Texas, USA

**Keywords:** Dental implants, dental prosthesis, implant-supported, dental abutments, biomechanics, prosthesis failure.

## Abstract

**Background:**

Mechanical stability is a critical factor for the long-term success of implant-supported rehabilitations. The maintenance of preload within the implant-abutment complex plays a fundamental role in preventing prosthetic complications, particularly screw loosening and loss of joint stability. Advances in monitoring technologies, including ultrasonic methods, piezoelectric sensors, and electromechanical impedance techniques, have introduced new possibilities for the early detection of biomechanical alterations in implant-supported prosthetic components.

**Materials and Methods:**

A structured narrative review was performed through comprehensive literature searches in PubMed/MEDLINE, Scopus, Web of Science and SciELO databases. The retrieved literature was critically analyzed according to biomechanical principles, preload maintenance, screw loosening mechanisms and emerging monitoring technologies.

**Results:**

The reviewed studies demonstrated that preload maintenance is essential for the biomechanical stability and longevity of implant-supported rehabilitations. Prosthetic screw loosening was identified as a prevalent mechanical complication, especially under cyclic and non-axial loading conditions. Internal conical and Morse taper connections generally showed improved preload maintenance compared with external hexagon systems. Additionally, ultrasonic monitoring, piezoelectric sensors, and electromechanical impedance methods demonstrated potential for early detection of preload loss and biomechanical instability. Although promising, most monitoring technologies remain limited to experimental investigations.

**Conclusions:**

Mechanical stability in implant-supported rehabilitations is strongly associated with preload preservation and implant-abutment integrity. Advanced monitoring technologies show promising potential for improving the early diagnosis of biomechanical instability; however, further prospective clinical studies are required before routine clinical implementation can be recommended.

## Introduction

The mechanical stability of implant-supported connections is a key determinant of the clinical success and longevity of implant rehabilitations. Prosthetic screws and implant-abutment interfaces play a critical role in maintaining preload, ensuring adequate stress distribution, and preserving the biomechanical integrity of the implant-supported system. Loss of preload and screw loosening are among the most common mechanical complications associated with implant-supported prostheses and may lead to micromovement, bacterial infiltration, prosthetic failure, and peri-implant bone loss. Previous studies have demonstrated that implant-abutment stability is closely related to preload maintenance and the mechanical behavior of prosthetic screws under functional loading conditions [1-4].

Contemporary implant-supported rehabilitations frequently employ internal conical and Morse taper connections because of their favorable biomechanical characteristics and enhanced resistance to micromovement. In these systems, the prosthetic abutment is secured to the implant by a fixation screw that generates preload during torque application. This preload creates a clamping force that promotes intimate contact between the implant and abutment surfaces, increasing frictional retention and joint stability. However, preload reduction over time may compromise the integrity of the implant-abutment interface and increase the risk of screw loosening and prosthetic instability [1,5].

Accurate assessment of preload is essential for the long-term stability of implant-supported restorations. Clinically, preload is estimated indirectly through tightening torque; however, torque does not represent a direct measurement of clamping force. Factors such as thread friction, manufacturing tolerances, surface wear, torque wrench inaccuracies, and thread misalignment may significantly affect the relationship between applied torque and the resulting preload [3,5]. Consequently, identical torque values may generate different clamping forces among implant systems and clinical situations.

In engineering applications, several methods have been developed to directly evaluate preload in bolted joints. Among these, ultrasonic measurement of screw elongation has emerged as a promising technique. Based on the acoustoelastic principle, ultrasonic methods correlate changes in wave propagation characteristics with variations in axial stress within the screw, allowing indirect assessment of preload conditions [3,6]. Because preload is directly related to screw elongation, ultrasonic approaches may provide greater accuracy than conventional torque-based measurements. The adaptation of such technologies to Implant Dentistry could enable early detection of preload loss and improve the monitoring of implant-supported prosthetic stability.

The mechanisms responsible for screw loosening in implant-supported prostheses are comparable to those observed in industrial bolted joints, where maintenance of clamping force is essential for structural stability. Inadequate preload, mechanical fatigue, plastic deformation, vibration, and cyclic loading have been identified as major contributors to joint instability over time [3]. These factors are particularly relevant in Implant Dentistry because prosthetic components are continuously exposed to complex and repetitive masticatory forces [3,4].

Prosthetic screw loosening remains one of the most prevalent mechanical complications in implant-supported prostheses, especially in single crowns and restorations subjected to non-axial loading. Factors such as prosthetic misfit, implant connection design, component geometry, and tightening torque directly influence the mechanical stability of the implant-abutment assembly [5,7]. Furthermore, the settling effect occurring shortly after torque application promotes preload reduction, supporting the recommendation of retorque protocols during prosthetic installation [8].

Recent advances in engineering have introduced novel methods for monitoring clamping force and detecting joint loosening, including ultrasonic techniques, electromechanical impedance analysis, piezoelectric sensors, and vibration-based approaches [3,9]. These technologies offer potential applications in Implant Dentistry, particularly for the real-time assessment of prosthetic stability and early detection of biomechanical alterations. Studies have shown that ultrasonic monitoring may identify changes in preload and screw stability, while piezoelectric and impedance-based systems can detect variations in contact stiffness and mechanical integrity [3,6,10-13].

Contact stiffness is another important factor influencing the stability of implant-supported restorations. Alterations in contact area and energy dissipation between connected surfaces are directly associated with preload maintenance and mechanical performance [3,13]. In implant-supported systems, passive fit among the implant, abutment, and prosthetic component is essential to minimize stress concentration and reduce the risk of mechanical complications. Therefore, understanding the biomechanical behavior of implant-abutment interfaces may contribute to the development of more stable and predictable restorative solutions [14].

Despite advances in implant biomechanics and preload maintenance, the potential application of non-invasive monitoring technologies in Implant Dentistry has not been comprehensively addressed. This review aims to integrate established biomechanical principles with emerging monitoring strategies while critically evaluating their limitations and clinical applicability. Although these technologies have shown promising results in engineering fields, evidence supporting their use in Implant Dentistry remains limited. The development of reliable, non-invasive methods for early detection of biomechanical instability may improve preventive diagnostics, optimize maintenance protocols, and enhance the longevity of implant-supported rehabilitations. Therefore, this study critically reviews the biomechanical factors influencing implant-supported prosthetic stability and explores the potential role of advanced monitoring technologies in Implant Dentistry.

## Materials and Methods

*Study Design and Literature Search Strategy*

This narrative review was conducted to critically discuss the biomechanical factors associated with the stability of implant-supported prosthetic components and the emerging technologies proposed for monitoring preload maintenance and screw loosening. A structured literature search was performed in the PubMed/MEDLINE, Scopus, Web of Science, and SCIELO databases to identify publications relevant to implant biomechanics, implant-abutment connections, preload maintenance, prosthetic screw loosening, and monitoring technologies, including ultrasonic, piezoelectric, electromechanical impedance, and vibration-based methods.

Both classical landmark studies and recent publications were considered to provide a comprehensive overview of the current evidence. The retrieved literature was analyzed qualitatively according to its scientific relevance, methodological consistency, clinical applicability, and contribution to understanding the biomechanical behavior of implant-supported prosthetic components. Because this manuscript is a narrative review, the objective was not to systematically identify all available evidence, but rather to critically synthesize representative studies that support the main concepts discussed throughout the manuscript.

*Selection of Representative Literature*

Representative publications were selected according to their relevance to implant biomechanics, preload maintenance, prosthetic screw loosening, and monitoring technologies. Preference was given to landmark studies, systematic reviews, clinical investigations, and recent publications that contributed to the understanding of the topics discussed in this review. Studies not directly related to implant-supported prosthetic stability or monitoring technologies were not considered.

*Data Synthesis*

The selected studies were qualitatively synthesized and organized according to the main thematic domains of the review, including implant-abutment mechanical stability, preload maintenance, biomechanical behavior of prosthetic interfaces, ultrasonic monitoring, piezoelectric sensors, electromechanical impedance methods, and vibration-based monitoring technologies.

To facilitate the presentation of the literature discussed throughout this review, Table 1 summarizes representative studies that illustrate the principal biomechanical concepts, monitoring approaches, and their potential clinical relevance (15-27). The studies were selected for their historical importance, scientific relevance, and contribution to the understanding of implant-supported prosthetic stability rather than as part of a systematic evidence synthesis.

## Results

*Implant-Abutment Mechanical Stability*

Mechanical stability is considered one of the main determinants of long-term success in implant-supported rehabilitations, since the implant-abutment interface is continuously subjected to axial, lateral, and cyclic loading forces that may compromise prosthetic integrity over time [1,7,28]. The maintenance of preload within the prosthetic screw joint is essential to ensure adequate clamping force and minimize micromovements at the implant-abutment interface [5,8]. Inadequate preload may result in screw loosening, prosthetic instability, bacterial infiltration, and peri-implant bone loss [15,26,28].

Previous studies demonstrated that prosthetic screw loosening remains one of the most frequent mechanical complications associated with implant-supported prostheses, especially in single-unit restorations and prostheses exposed to non-axial loading conditions [15,17,18]. Goodacre *et al*. [26] reported that screw loosening represents one of the most prevalent technical complications in implant prosthodontics, whereas Theoharidou *et al*. [15] observed that connection design directly influences complication rates. Internal conical and Morse taper connections demonstrated improved mechanical stability and reduced micromovement compared with external hexagon systems [16,22]. However, although Morse taper connections may reduce screw loosening incidence, clinical complications may still occur under excessive occlusal loading or inadequate prosthetic adaptation [22,23].

*Preload Maintenance and Prosthetic Screw Loosening*

Preload corresponds to the tensile force generated after torque application, creating clamping force between the implant and prosthetic component [1,5]. The literature consistently demonstrates that preload maintenance is fundamental for prosthetic stability; however, preload loss may occur due to settling effect, cyclic fatigue, vibration, and plastic deformation of contacting surfaces [3,9,17].

Siamos *et al*. [9] demonstrated that preload reduction significantly increases the risk of screw loosening under cyclic loading conditions. Similarly, Pardal-Peláez and Montero [17] reported that dynamic fatigue promotes progressive preload loss regardless of implant connection type. Internal conical and Morse taper systems generally demonstrated lower loosening rates and better preload maintenance than external hexagon connections [16,22,29]. Nevertheless, Pitman *et al*. [18] observed that angulated restorations may still present increased mechanical complications even in conical connections due to unfavorable stress distribution.

The magnitude of applied torque, screw design, lubrication, and manufacturing precision also directly affect preload maintenance and biomechanical performance [5,8,30]. Mohammed *et al*. [29] demonstrated that screw length and cyclic loading significantly influence removal torque values, especially in external hex systems. In addition, finite element analyses suggested that transversal screw designs may increase stress concentration and mechanical complication risk under loading conditions [31].

*Biomechanics of Implant-Abutment Interfaces*

The biomechanical behavior of implant-supported prostheses is strongly dependent on the precision of fit at the implant-abutment interface [19,27]. Microscopic discrepancies between contacting surfaces may alter stress distribution, reduce contact stiffness, and increase energy dissipation, contributing to prosthetic instability [19,20].

Binon [27] demonstrated that implant-abutment misfit negatively affects screw joint stability and increases micromovement under loading conditions. Similarly, Kano *et al*. [19] proposed a classification system for evaluating implant-abutment microgaps, emphasizing their influence on biomechanical behavior. Tiossi *et al*. [20] observed that cyclic loading may increase vertical microgaps in implant-supported prostheses, potentially compromising long-term stability.

From a biological perspective, Boynueğri *et al*. [21] demonstrated that microgap localization may influence inflammatory cytokine levels in peri-implant crevicular fluid, suggesting that biomechanical instability may also contribute to peri-implant inflammatory responses. Therefore, precise manufacturing and passive prosthetic fit remain essential for reducing mechanical and biological complications [16,21,27].

*Ultrasonic Monitoring and Acoustoelastic Principles*

Ultrasonic monitoring methods based on acoustoelastic principles have emerged as promising non-destructive techniques for evaluating preload maintenance in bolted systems [3,6]. These methods correlate ultrasonic wave propagation with variations in axial stress within the screw structure, allowing indirect evaluation of preload conditions [3].

Guo *et al*. [6] demonstrated that acoustic resonance frequency analysis may effectively detect prosthetic screw instability in implant-supported fixed prostheses under laboratory conditions. Compared with conventional torque assessment methods, ultrasonic monitoring may provide earlier detection of preload loss and micromovement [3,6]. However, limitations related to signal interpretation, intraoral environmental interference, saliva contamination, and component geometry still restrict clinical applicability [6].

Although engineering studies demonstrated high sensitivity of ultrasonic approaches for loosening detection [3,12], evidence in Implant Dentistry remains limited predominantly to in vitro investigations. Therefore, additional clinical studies are required before widespread clinical implementation can be recommended, (Fig. 1).

*Piezoelectric Sensors and Electromechanical Impedance*

Piezoelectric sensors and electromechanical impedance (EMI) methods have gained attention for structural health monitoring due to their high sensitivity for detecting mechanical alterations and contact stiffness variations [24,25,32]. These systems operate through piezoelectric transducers capable of functioning simultaneously as sensors and actuators, enabling detection of vibrational and impedance changes associated with preload loss [25,32]. Despite engineering advances, clinical translation into oral rehabilitation remains challenging.

Studies in engineering applications demonstrated that EMI techniques may accurately identify loosening conditions in bolted structures [24,25]. Na and Baek [25] emphasized that piezoelectric impedance methods provide high sensitivity for detecting early mechanical instability, whereas Huynh *et al*. [24] reported that smart piezoelectric interfaces may improve monitoring reliability. However, most available evidence originates from structural engineering applications rather than oral rehabilitation models [24,32,33].

Although these technologies present promising potential for Implant Dentistry, important clinical limitations remain, including miniaturization requirements, sensor durability, intraoral humidity, and long-term biocompatibility [32,34,35]. Therefore, their clinical applicability in implant-supported prostheses should still be interpreted cautiously.

*Vibration-Based and Nonlinear Dynamic Methods*

Vibration-based monitoring systems and nonlinear dynamic analyses have also been proposed for evaluating loosening conditions in bolted joints [11,12,14]. These methods are based on the detection of resonance frequency shifts, harmonic responses, and vibroacoustic modulation generated by changes in interface stiffness [12,14].

Pineda Allen and Ng [12] demonstrated that nonlinear guided-wave mixing may identify loosening conditions with high sensitivity in structural joints. Similarly, Li *et al*. [14] reported that digital image correlation methods may allow early bolt loosening detection through dynamic deformation analysis. Although these approaches demonstrated promising sensitivity in engineering applications, evidence regarding their application in Implant Dentistry remains scarce.

The incorporation of machine learning and pattern-recognition algorithms has been suggested to improve diagnostic sensitivity [11,14]. However, these approaches are still predominantly experimental, and current evidence does not support direct clinical application in implant-supported prostheses. Consequently, further validation studies are necessary before these technologies can be translated into routine clinical practice.

*Future Perspectives in Implant Prosthetic Monitoring*

Recent advances in biomechanical monitoring suggest that non-invasive technologies may eventually improve the diagnosis and maintenance of implant-supported rehabilitations [3,6,24]. The integration of ultrasonic analysis, piezoelectric sensors, and electromechanical impedance systems may contribute to earlier identification of preload loss and prosthetic instability [6,24,25].

However, despite promising laboratory findings, most available studies remain experimental and lack long-term clinical validation [6,24,32,33]. Factors such as oral humidity, thermal variation, masticatory loading complexity, and reduced prosthetic dimensions still represent important limitations for clinical implementation. Therefore, although smart monitoring technologies may become relevant adjunctive tools in Implant Dentistry, current evidence remains insufficient to support routine clinical application.

## Discussion

The present review highlights that the long-term success of implant-supported rehabilitations depends not only on osseointegration, but also on the mechanical stability of prosthetic components and the preservation of preload over time [1,5,15]. Although prosthetic screw loosening remains a common complication, its occurrence appears to be multifactorial and strongly influenced by the interaction among implant connection design, torque application, prosthetic fit, and occlusal loading conditions [15,17,26]. Clinical evidence suggests that internal conical and Morse taper connections provide improved mechanical stability compared with external hexagon systems due to enhanced frictional engagement and reduced interface micromovement [16,22]. Nevertheless, even conical connections may present biomechanical complications when associated with unfavorable loading distribution or prosthetic misfit [18,23].

Recent investigations have further strengthened current evidence regarding preload maintenance and prosthetic screw stability. Guo *et al*. [6] demonstrated that acoustic resonance frequency analysis can accurately detect prosthetic screw instability in implant-supported fixed dental prostheses under laboratory conditions, reinforcing the potential of non-invasive monitoring approaches. Likewise, Bishti *et al*. [32] demonstrated that implant design, prosthetic component geometry, screw characteristics, and tightening protocols significantly influence preload maintenance and long-term screw stability in implant-supported single crowns. Complementing these findings, Pitman *et al*. [18] showed that angulation-correcting restorations are associated with a higher risk of screw loosening due to unfavorable biomechanical loading, whereas Szajek and Wierszycki [10] demonstrated that preload loss under cyclic occlusal loading is a reliable predictor of future loosening events. Collectively, these recent studies reinforce the biomechanical concepts discussed in the present review while emphasizing the need for further clinical validation of monitoring technologies.

Another important aspect identified in the literature is the direct relationship between microgap formation and biological complications. In addition to affecting preload maintenance, implant-abutment misfit may facilitate bacterial infiltration and inflammatory reactions within peri-implant tissues [19,21]. Therefore, biomechanical instability should not be interpreted exclusively as a technical complication, since it may also influence peri-implant tissue health and compromise long-term treatment success. This finding reinforces the importance of accurate manufacturing processes and passive prosthetic adaptation in implant-supported rehabilitations [20,27].

The reviewed studies also demonstrated that cyclic loading and settling effect significantly reduce preload shortly after torque application [3,9]. From a clinical perspective, these findings support the recommendation of retorque procedures during prosthetic installation and follow-up appointments to minimize mechanical instability [8,29]. In addition, the magnitude of tightening torque should be carefully controlled, since insufficient torque may reduce clamping force while excessive torque may increase stress concentration and risk of screw fracture [5,30]. Consequently, adherence to manufacturer-recommended torque values remains essential for maintaining prosthetic stability and reducing long-term complications.

Another relevant consideration involves the increasing use of angulated abutments and complex prosthetic rehabilitations in contemporary Implant Dentistry. Although these approaches improve prosthetic versatility and esthetics, they may also alter stress distribution and increase mechanical complications under non-axial loading conditions [18,23,31]. Finite element analyses demonstrated that stress concentration tends to increase in prosthetic screws and implant interfaces when loading vectors are unfavorable, potentially accelerating preload loss and component fatigue [31]. Therefore, prosthetic planning should consider not only esthetic demands, but also biomechanical behavior and long-term maintenance requirements.

Recent advances in biomechanical monitoring technologies may provide valuable adjunctive tools for preventive maintenance in Implant Dentistry. Ultrasonic methods, piezoelectric sensors, and electromechanical impedance techniques demonstrated high sensitivity for detecting alterations in preload and contact stiffness in engineering applications [3,6,24,25]. However, despite promising laboratory findings, the translation of these technologies into clinical practice remains limited by several factors, including reduced prosthetic dimensions, intraoral humidity, thermal variation, and the complexity of masticatory loading patterns [24,32,33]. Furthermore, current evidence remains predominantly experimental, with limited clinical validation in implant-supported prostheses.

Although ultrasonic, piezoelectric, vibration-based and electromechanical impedance techniques have demonstrated excellent sensitivity under controlled laboratory conditions, several important barriers still limit their translation into routine clinical practice. Sensor miniaturization, long-term durability, resistance to intraoral humidity, thermal fluctuations, prosthetic geometry, patient-specific occlusal variability, and long-term biocompatibility remain significant technological challenges. Furthermore, most currently available evidence originates from engineering models and laboratory investigations rather than prospective clinical studies. Consequently, the diagnostic potential of these technologies should be interpreted cautiously until robust clinical validation confirms their accuracy, reproducibility, and cost-effectiveness in Implant Dentistry. From a clinical perspective, clinicians should prioritize passive prosthetic fit, strict adherence to manufacturer-recommended torque values, retorque protocols after settling, and periodic maintenance appointments. Emerging monitoring technologies may eventually support early diagnosis of preload loss; however, at present they should be regarded as complementary research tools rather than routine clinical methods.

The incorporation of artificial intelligence and pattern-recognition algorithms into monitoring systems has also been proposed as a potential strategy for improving diagnostic sensitivity and predictive analysis of mechanical complications [11,14]. Nevertheless, the available evidence is still preliminary and insufficient to support routine clinical implementation. Therefore, future investigations should prioritize prospective clinical studies capable of validating the accuracy, reproducibility, and long-term reliability of these technologies in oral rehabilitation settings. Among the evaluated monitoring approaches, ultrasonic methods demonstrated greater applicability for preload assessment, whereas piezoelectric and vibration-based systems remain predominantly experimental.

Unlike previous reviews focusing exclusively on screw loosening or preload maintenance, the present review integrates classical biomechanical concepts with emerging monitoring technologies, highlighting both current evidence and the challenges that continue to limit their routine clinical implementation.

This review has several limitations that should be acknowledged. The heterogeneity among the included studies, including variations in implant systems, loading protocols, and biomechanical methodologies, limits direct comparison of findings. In addition, much of the available evidence on advanced monitoring technologies originates from laboratory or engineering-based investigations rather than clinical implant research, restricting direct clinical extrapolation. The narrative design of this review may also introduce selection bias and subjective interpretation. Therefore, further well-designed clinical studies and systematic reviews are required to establish standardized protocols and confirm the clinical applicability of advanced biomechanical monitoring systems in Implant Dentistry. Clinically, emphasis should remain on passive fit, appropriate torque application, and regular maintenance to minimize long-term prosthetic complications.

Despite these limitations, this review contributes by integrating established concepts of preload maintenance with recent advances in structural health monitoring. By critically evaluating ultrasonic, piezoelectric, electromechanical impedance, and vibration-based technologies, it highlights current evidence gaps and identifies priorities for future translational research aimed at improving early detection of prosthetic complications.

## Conclusions

Current evidence supports the importance of preload maintenance for preserving implant-supported prosthetic stability. Emerging monitoring technologies such as ultrasonic analysis, piezoelectric sensing and electromechanical impedance demonstrate promising diagnostic potential, although their application remains predominantly experimental. Further prospective clinical studies are required before these technologies can be incorporated into routine implant practice.

## Figures and Tables

**Table 1:** Summary of representative publications illustrating the principal biomechanical concepts, preload maintenance mechanisms, and monitoring technologies discussed in this narrative review.

**Author / Year**	**Publication Type**	**Main Objective**	**Main Approach**	**Main Findings**	**Clinical Implications**
Haack *et al*., 1995 [5]	*In vitro*	Evaluate preload stress in implant screws	Torque/preload analysis	Preload directly influenced screw stability	Reinforces the importance of adequate torque application for maintaining prosthetic stability
Tan & Nicholls, 2001 [8]	Experimental	Compare preload among abutment systems	Torque maintenance analysis	Different implant systems showed variable preload maintenance	Demonstrates that implant connection design influences long-term mechanical performance
Siamos *et al*., 2002 [9]	*In vitro*	Assess preload loss under cyclic loading	Cyclic fatigue simulation	Preload reduction increased screw loosening risk	Supports clinical retorque protocols after prosthetic installation
Theoharidou *et al*., 2008 [15]	Systematic review	Evaluate abutment screw loosening	Literature review	Connection design influenced loosening rates	Highlights the importance of selecting stable implant-abutment connections
Gracis *et al*., 2012 [16]	Systematic review	Compare internal vs external connections	Systematic analysis	Internal conical connections demonstrated improved stability	Suggests conical connections may reduce mechanical complications
Pardal-Peláez & Montero, 2017 [17]	Systematic review	Analyze preload loss after fatigue	Dynamic fatigue evaluation	Cyclic loading promoted progressive preload loss	Reinforces the need for periodic maintenance and monitoring
Pitman *et al*., 2024 [18]	Systematic review	Evaluate screw loosening in angulated restorations	In vitro studies review	Angulated restorations increased biomechanical complications	Indicates increased mechanical risk in non-axial prosthetic rehabilitations
Kano *et al*., 2007 [19]	Experimental	Classify implant-abutment microgaps	Microscopic analysis	Misfit influenced biomechanical behavior	Demonstrates the importance of passive fit in preventing instability
Tiossi *et al*., 2014 [20]	*In vitro*	Evaluate cyclic loading on zirconia frameworks	Fatigue loading	Cyclic loading increased vertical microgaps	Suggests long-term loading may compromise prosthetic adaptation
Boynueğri *et al*., 2012 [21]	Clinical study	Analyze biological effects of microgaps	Cytokine evaluation	Microgap localization influenced peri-implant inflammation	Demonstrates the relationship between biomechanical instability and peri-implant health
Mangano *et al*., 2009 [22]	Prospective clinical study	Evaluate Morse taper implants	Clinical follow-up	Morse taper connections showed favorable stability	Supports the clinical use of Morse taper systems for improved stability
Omori *et al*., 2020 [23]	Systematic review	Analyze complications of angulated abutments	Meta-analysis	Angulated abutments increased mechanical complications	Reinforces careful prosthetic planning in angulated rehabilitations
Guo *et al*., 2025 [6]	*In vitro*	Evaluate acoustic resonance monitoring	Ultrasonic analysis	Ultrasonic methods detected screw instability	Suggests potential for non-invasive monitoring of preload loss
Miao *et al*., 2020 [3]	Narrative review	Review bolt loosening detection methods	Engineering-based review	Ultrasonic and vibration methods showed high sensitivity	Provides biomechanical concepts potentially applicable to Implant Dentistry
Huynh *et al*., 2020 [24]	Experimental	Evaluate piezoelectric monitoring systems	Electromechanical impedance	Smart interfaces improved monitoring sensitivity	Demonstrates potential for early detection of biomechanical instability
Na & Baek, 2018 [25]	Review	Review EMI monitoring techniques	Structural health monitoring	Piezoelectric impedance methods showed high sensitivity	Suggests EMI methods may improve preventive prosthetic monitoring
Pineda Allen & Ng, 2021 [12]	Experimental	Evaluate nonlinear guided-wave monitoring	Wave mixing analysis	Nonlinear vibration methods identified loosening conditions	Demonstrates high sensitivity for detecting early mechanical alterations
Li *et al*., 2024 [14]	Experimental	Detect bolt loosening using image correlation	Digital image correlation	Dynamic deformation analysis detected early instability	Indicates potential applications for biomechanical monitoring systems
Goodacre *et al*., 2003 [26]	Clinical review	Evaluate implant complications	Literature review	Screw loosening was one of the most frequent complications	Emphasizes the clinical relevance of mechanical complication prevention
Binon, 1996 [27]	Experimental	Analyze implant-abutment misfit	Hexagonal misfit evaluation	Misfit negatively affected joint stability	Reinforces the importance of precision manufacturing and passive fit

**Figure 1:**
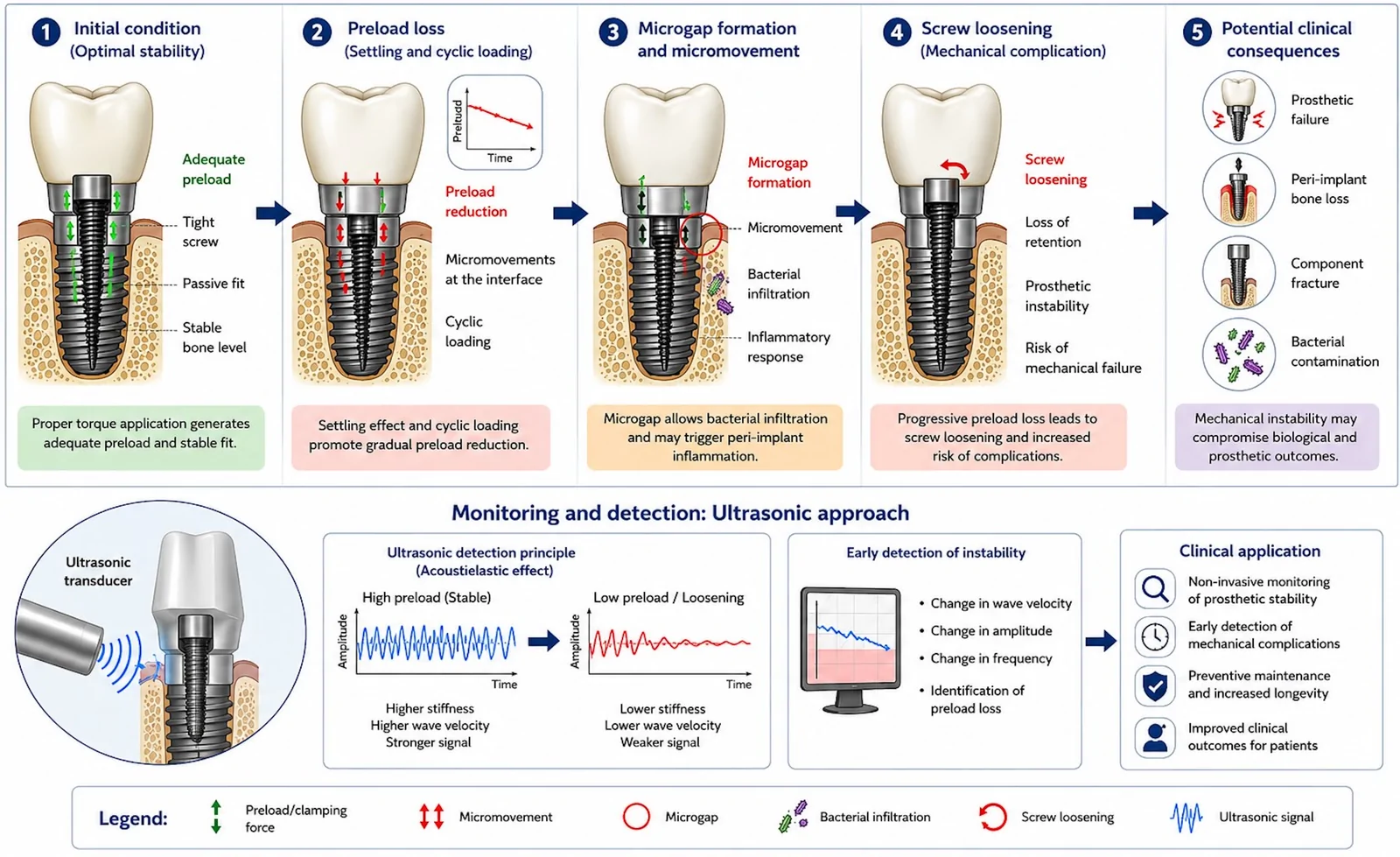
Schematic illustration of preload loss, microgap formation, prosthetic screw loosening, and ultrasonic monitoring approaches in implant-supported prosthetic components.

## Data Availability

The datasets generated and analyzed during the current study are available from the corresponding author upon reasonable request.
